# Fibular head transfixion wire and its relationship to common peroneal nerve: cadaveric analysis

**DOI:** 10.1007/s11751-015-0225-3

**Published:** 2015-05-28

**Authors:** Paul Dearden, Kathryn Lowery, Kevin Sherman, Vishy Mahadevan, Hemant Sharma

**Affiliations:** Hull Royal Infirmary, Anlaby Road, Hull, UK; Royal College of Surgeons, England, UK

**Keywords:** Ilizarov, Peroneal nerve, Anatomy, Frame, Proximal tibial fractures, Limb lengthening

## Abstract

Proximal tibio-fibular joint is routinely stabilised during leg lengthening, peri-articular fractures and deformity corrections of tibia. Potential injury to the common peroneal nerve at the level of the fibula head/neck junction during wire insertion is a recognised complication. Previous studies have mapped the course of the common peroneal nerve and its branches at the level of the fibular head, and guidelines are published regarding placement of proximal tibial wires. This study aims to relate the course of the common peroneal nerve to the placement of a lateral insertion fibula head transfixion wire. Standard 1.8-mm Ilizarov ‘olive’ wires were inserted in the fibula head of 10 un-embalmed cadaveric knees. Wires were inserted percutaneously to the fibula head using surface anatomy landmarks and palpation technique. The course of the common peroneal nerve was then dissected. Distances from wire entry point to the course of the common peroneal nerve were measured post-wire insertion. The mean distance of the common peroneal nerve from the anterior aspect of the broadest point of the fibular head was 24.5 mm (range 14.2–37.7 mm). Common peroneal nerve was seen to cross the neck of fibula at a mean distance of 34.8 mm from the tip of fibula (range 21.5–44.3 mm). Wire placement was found to be on average, 52 % of the maximal AP diameter of the fibula head and 64 % of the distance from tip of fibula to the point of nerve crossing fibula neck. When inserting a fibula head transfixion wire, care must be taken not to place wire entry point too distal or posterior on the fibula head. Observing a safe zone in the anterior half of the proximal 20 mm of the fibula head would avoid injury to the nerve. In cases where palpation of fibula is difficult due to patient habitus, we recommend consideration of the use of fluoroscopic guidance during wire transfixion of the proximal tibio-fibular articulation to avoid wire insertion too distally and subsequent potential nerve injury.

## Introduction

Fine wire circular frame fixation for tibial fractures is commonly utilised in the trauma setting, deformity corrections and leg lengthening. In proximal tibial fractures/severe plateau fractures, there is lack of space and fibular head provides not only space and but decent spread of wires, thereby increasing the crossing angle of wires. During leg lengthening and deformity corrections, the fibula has to be transfixed at both ends to prevent any subluxation of either tibio-fibular joint.

Placement of the proximal wires from the lateral side is commonly performed using palpation techniques and observation of Ilizarov ‘safe corridors’. The wires are passed percutaneously, without visualisation of the underlying peroneal nerve. Commonly, wires are passed without radiographic imaging, or with the focus of imaging being on the orientation of wires parallel to the knee articular surface. Injury to the peroneal nerve can have devastating consequences, and its iatrogenic occurrence during external fixation surgery to the proximal tibia has been recognised [1, 2]. The common peroneal nerve supplies motor innervation to the muscles of dorsiflexion of the foot and extension of the toes, and therefore, injury can have a significant effect on the patients’ gait. Previous studies have described the anatomy and course of the peroneal nerve and the relationship to the proximal tibia [3–5]. Ruben et al. [6] studied the relationship of the peroneal nerve to Gerdy’s tubercle and determined a safe zone for the insertion of proximal tibial wires. Our study aimed to measure the distance of the nerve from a wire inserted into the proximal fibula percutaneously reproducing the clinical environment and to attempt to provide recommendations for the safe placement of wires passed from the lateral aspect into the proximal tibia through the fibula head.

## Materials and methods

Ten cadaveric knee specimens were obtained. The specimens were fresh and un-embalmed allowing dissection of the nerve with no concern for the condition of the soft tissues altering the measurements. As the knee specimens had not undergone any form of embalming or tissue ‘fixation’, the specimens handled in a very similar way to normal healthy tissue with regard to range of movement with no discernible stiffness. The specimens were sectioned knee joints and had been removed from the cadaver at approximately mid-femur and mid-tibia. The details of the specimen in regard to demographics of the donor such as age, sex or height were unknown.

**Fig. 1:**
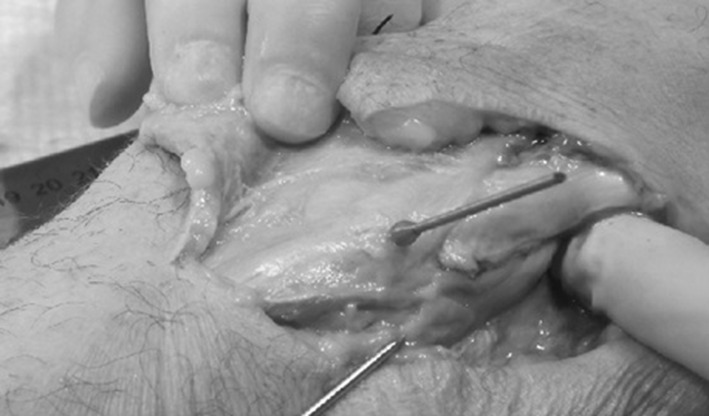
Demonstrating the lateral approach centred on the fibula. The photograph shows the wire in the fibula and the nerve lying posteriorly to the fibula

**Fig. 2:**
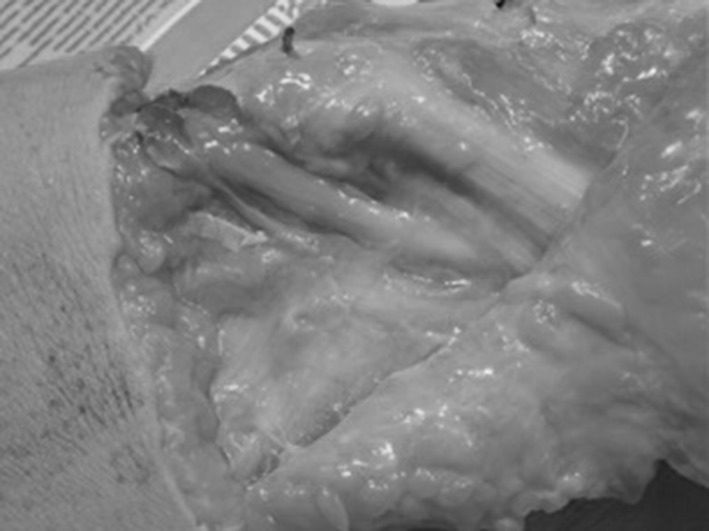
Demonstration of the nerve released at point which it crosses the fibula neck but the attachments are not released to obtain accurate measurements

The knees were positioned in slight flexion of approximately 30° and in neutral rotation. Standard 1.8-mm Ilizarov fibula head olive wires were placed to transfix the fibula head to the proximal tibia. The wires were inserted by two orthopaedic registrars, five specimens each, under the supervision of a consultant surgeon experienced with the insertion of such wires in circular frame surgery. This ensured that any bias related to position of wires related to the surgeons experience with wire insertion was reduced. The surface markings of the fibula head were palpated and the wire inserted aiming to be in the centre of the head in both planes. Following insertion, a lateral dissection centred on the fibula was carried out, with the knee remaining slightly flexed. The nerve was identified proximally as it passes posterior to the lateral head of gastrocnemius and gently dissected along its course toward the fibula head, Fig. 1. Care was taken not to release any soft tissue attachments of the nerve to allow its true anatomical position and course to be as closely maintained as possible during dissection, Fig. 2. Measurements were carefully taken to avoid disturbance of the course of the nerve, if further dissection was required to expose a relevant anatomical point; measurements were taken before further dissection occurred. Measurements were taken using Vernier electronic digital callipers.

Measurements recorded were the point at which the nerve crosses the fibula neck in relation to the distance from the tip of the styloid process of the fibula, the distance from the anterior aspect of the fibula head at its broadest point to the position of the nerve lying posterior to the fibula and the diameter of the fibula at its broadest point. The position of the inserted wire was then analysed in relation to the tip of styloid process of the fibula, the anterior aspect of the fibula and the distance to the nerve as it crosses the fibula neck. These anatomical landmarks were defined following direct visualisation after dissection. From these measurements, ratios were calculated to create guidelines for safe zones of wire insertion to avoid injury to the nerve.

## Results

**Fig. 3:**
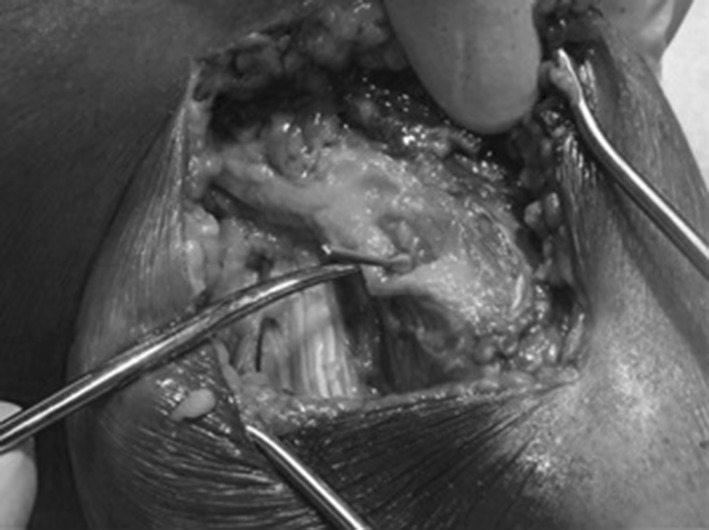
Demonstration of the specimen in which the wire had been inserted posteriorly and distally near the fibula neck. The wire was touching the peroneal nerve as it was lying at the posterior edge of the fibula; however, although slightly indenting the nerve, it was not penetrating it. *The discolouration* seen in the specimen in this picture is from a previous study which had injected dye into the knee joint and dye had diffused into surrounding tissues. The previous study had no effect on the results of this study (colour figure online)

The mean diameter of the fibula at its widest point was 22.3 mm (range 18.3–29.9 mm). The wire was inserted on average 11.8 mm from the anterior aspect of the fibula (range 5.5–15.4 mm), Table 1. The mean distance of the common peroneal nerve from the anterior aspect of the broadest point of the fibular head was 24.5 mm (range 14.2–37.7 mm), Table 1. From these measurements, a ratio was determined, thus demonstrating that, on average, the wire was inserted 52 % of the way back from the anterior edge of the fibula (range 24–77 %) in relation to its maximal AP diameter. Wires were on average 49 % of the distance from the anterior aspect of the fibula to the nerve lying posteriorly (range 26–100 %) (Table 1). In one specimen, the wire was inserted in such a way that it was noted to be touching the peroneal nerve in its location at the posterior edge of the fibula. Although the wire was seen to be slightly indenting the nerve, it was not found to be penetrating it, Fig. 3. In this specimen, a comment was recorded during wire insertion that ‘anatomical landmark palpation was particularly difficult in this specimen due to the adipose tissue and muscle bulk’.

**Table 1:** Average measurements in millimetres of the diameter of the fibula head at its maximum antero-posterior width, distances from the anterior aspect of the fibula to the wire, and to the nerve lying posteriorly

Specimen	AP fib head diameter (mm)	Anterior fibula to wire (mm)	Anterior fibula to nerve (mm)	Wire to nerve ratio (%)	Fib diameter/wire ratio (%)
1	29.89	15.24	37.77	40	51
2	22.83	5.51	21.33	26	24
3	22.53	6.49	23.69	27	29
4	21.83	11.8	22.71	52	54
5	22.25	10.84	20.89	52	49
6	20.54	7.79	20.44	38	38
7	21.28	14.17	28.16	50	67
8	20.49	12.49	27.35	46	61
9	23.41	15.35	28.56	54	66
10	18.35	14.21	14.21	100	77
Average	22.34	11.389	24.511	48.5	51.6

The last two columns show the ratios in percentages of the distance from the anterior fibula to the wire and to the nerve, and of the wire placement in the fibula head to its AP diameter

The distance from the tip of the styloid process of the fibula to the wire insertion point was on average 22.2 mm (range 13.5–32.3 mm) (Table 2). The distance from the tip of styloid process of the fibula to the nerve as it winds around the neck was 34.8 mm (range 21.5–44.3 mm) (Table 2). Calculating these results as a ratio, the wire was on average inserted 64 % of the distance from the fibula styloid to the point of nerve crossing fibula neck (range 43–100 %) (Table 2). The specimen which had the wire touching the nerve posteriorly was also touching the nerve as it crossed the fibula, having been inserted into the fibula at a far more distal point, just proximal to the fibula neck. As previously noted, it had not been easy to palpate the fibula head and upon dissection it would appear that the point which had been palpated was not the styloid process of the fibula as this structure is much deeper and posterior. The point palpated is suspected to have been the most prominent subcutaneous superior portion of the anterior border of the fibula.

**Table 2:** Average measurements in millimetres of the distance from the tip of the fibula head to the wire and to the nerve as it crosses the fibula neck and the percentage ratio of the distance of the wire from the tip of the fibula to the nerve

Specimen	Tip fibula to wire (mm)	Tip fibula to nerve (mm)	Ratio distance wire to nerve from tip fibula (%)
1	32.87	44.27	74
2	23.50	36.81	64
3	23.78	40.88	58
4	13.50	21.51	63
5	23.52	39.12	60
6	15.21	35.30	43
7	17.21	32.33	53
8	25.14	35.41	71
9	19.64	35.14	56
10	27.23	27.23	100
Average	22.16	34.80	64

## Discussion

Insertion of a lateral fibular head transfixion wire is often carried out using anatomical landmarks and palpation to plan placement. The surgeon aims to insert the wire in the centre of the fibula head in the sagittal and coronal plane. Our study examined the proximity of the wire to the common peroneal nerve. We have demonstrated that the distance from the tip of the styloid process of the fibula to the nerve as it winds around the neck was 34.8 mm (range 21.5–44.3 mm). This finding concurs with Rupp et al. [7] who demonstrated the nerve to wraps around the fibular neck at an average of 3.5 cm from the styloid process or tip (range 3–4.4 cm). Stitgen et al. [3] also studied the course and branches of the peroneal nerve at the level of the fibular neck; their study determined that a ‘safe zone’ from a line drawn between the fibular head and the tibial tuberosity superiorly and extending 2 cm distally minimised the risk of damage to the nerve. Rubel et al. determined an anatomical safe zone for wires inserted into the tibia from the lateral side based on Gerdy’s tubercle and mapping out a safe arc. However, these authors recommended that, in the presence of a fibula fracture, these dimensions may be altered and could only be observed reliably in an intact fibula [6]. The nature of the configuration of proximal and intra-articular tibial fractures which require circular frame surgery may often mean that the fibula is not intact. In these circumstances, location of the nerve in relation to fixed fibula landmarks may prove to be more helpful in determining a safe location in which to insert a wire.

The mean diameter of the fibular head at it broadest diameter in the AP plane was found to be 22.3 mm (range 18.3–29.9 mm). The wires in our series were seen to be inserted, on average, 11.8 mm from the anterior aspect of the fibula at its broadest point. From these measurements, a ratio was calculated and demonstrates that the wire was on average inserted in the centre or 52 % of total anterior-to-posterior diameter at its widest point (range 24–77 %) when using a landmark palpation technique. The distance of the nerve behind the fibula at the widest AP diameter was 24.5 mm (range 14.2–37.7 mm) from the anterior aspect of the fibula. In four of the ten specimens, the course of the nerve traversed the posterior border of the fibula head at its widest AP diameter. This is demonstrated in the discrepancy between the ranges demonstrated in AP diameter of the fibular head when compared to the position of the nerve in relation to the anterior border of the fibula head at its broadest point. As such, if the wire had been inserted too far posterior in the fibular head in these specimens, the nerve was at potentially increased risk of injury.

**Fig. 4:**
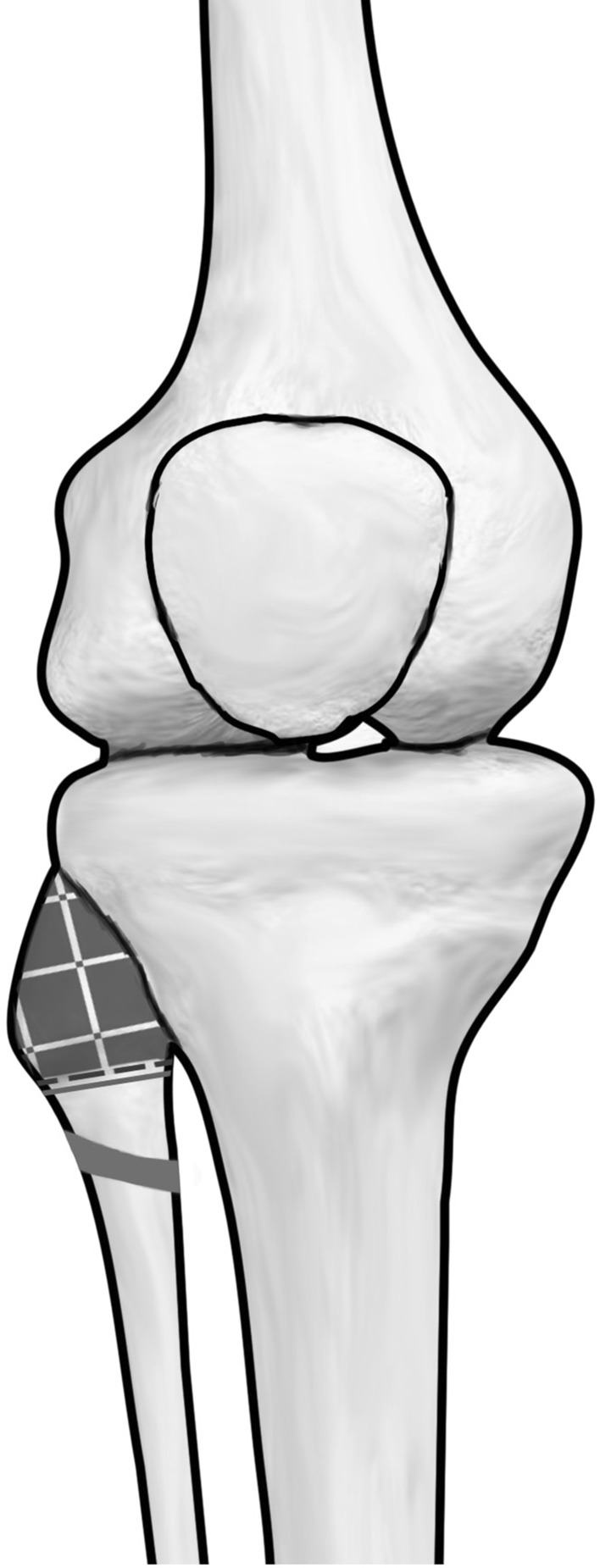
Illustration demonstrating the area recommended for insertion of the wire in the coronal plane. The *yellow lines* show the range of the nerve crossing the fibula neck measured from the tip of the fibula, range 21.5–44.3 mm. Based on the findings in this study, we recommend that proximal fibula head wires should be inserted no further than 2 cm distal to the tip of the fibular head and aim slightly anterior in the fibula head in sagittal plane at the level of its maximal diameter, (*patchwork area*) (colour figure online)

**Fig. 5:**
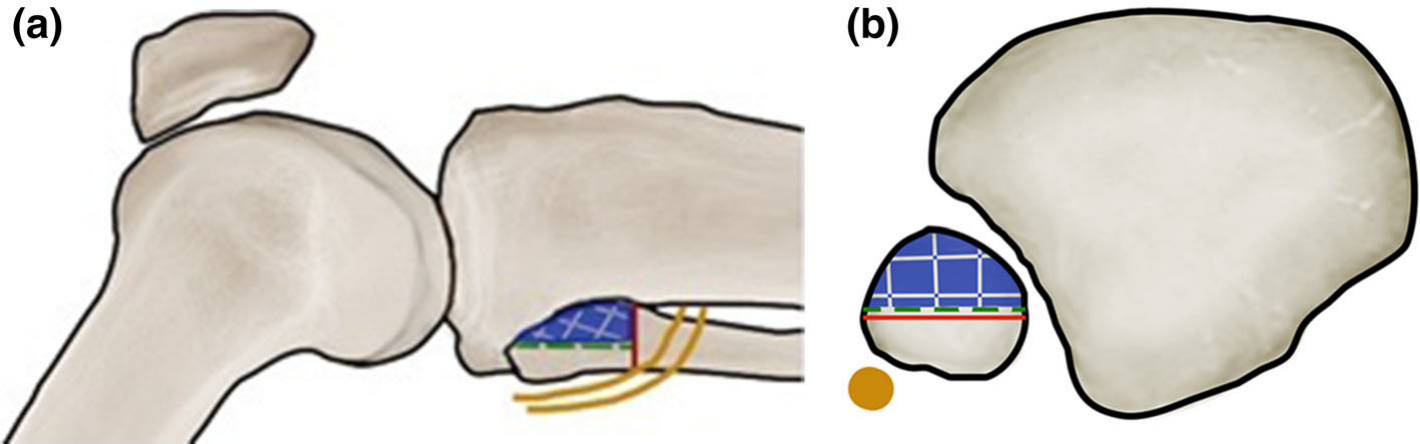
Illustration demonstrating the area recommended for insertion of the wire in the sagittal and axial planes

Calculating a ratio, the wires inserted in this study were 49 % of the distance from the anterior aspect of the fibula to the nerve lying posteriorly (range 26–100 %). However, in one specimen, the nerve lay touching the wire after insertion. In this specimen, the wire had been inserted more posteriorly in the fibula and had been inserted in a more distal position than in the other specimens, where the nerve begins to cross the neck of the fibula. After measurements had been taken, the fibula head was dissected fully in this specimen and was found to extend more proximally than had been felt on palpation. Therefore, using a landmark palpation technique, the wire was inserted too distally and too posterior risking injury to the common peroneal nerve. Following this finding, the authors recommend that, in cases in which the fibula head is easily palpable, surgeons should observe a safe zone located 2 cm from the tip of the styloid process of the fibula and within the anterior half of the fibula Figs. 4 and 5. The surgeon should consider using fluoroscopic guidance to locate this safe point prior to fibula transfixion wire insertion.

The authors recognise the limitations of this study. The cadaveric specimens used were not complete legs, and therefore, measurements of the length of the fibula were not possible. The measurements taken were expressed as ratios in relation to the maximal AP diameter of the fibula head and used to describe proximity of inserted wires to the common peroneal nerve.

In cases of trauma involving either tibial or fibula fracture, the local anatomy may be distorted as described by El-Shazly et al. Similarly, in cases of deformity, the bony anatomy and the course of the nerve around the proximal fibula may be distorted. The observations of this study only describe the anatomical location of the common peroneal nerve in specimens with normal proximal tibio-fibula anatomy and without fracture. In such cases, the position of the common peroneal nerve may be displaced during the initial trauma, especially when marked shortening of the tibia occurs [8], and it may be placed at additional risk during proximal fibula head wire insertion. Special care and consideration of open exposure of the common peroneal nerve may be required in such cases to avoid iatrogenic injury.

It was not possible to obtain information on the origin or demographic details of the specimens used in this study, and therefore, factors such as age or sex were not taken into account. The authors acknowledge that the findings of this study are of limited use in the paediatric population as none of the specimens dissected represented a skeletally immature individual. The anatomy in such cases may vary from that of the adult population and that limited inferences regarding the safe zone of wire insertion can be made from the finding of this paper. The authors appreciate that sample size in this study is small and that further studies allowing for larger numbers of male and female specimens are required to demonstrate any difference between the sexes in the course of the nerve and safe wire insertion zones if one exists.

Based on the findings in this study, we recommend that proximal fibula head wires should be inserted no further than 2 cm distal to the tip of the fibular head and aim slightly anterior in the fibula head in AP plane at the level of its maximal diameter, Fig. 4 and 5. Feeling the anterior edge of the fibula with wire and inserting the wire anteriorly will achieve correct placement of wire in AP plane. This will reduce the risk of placing the wire inadvertently too distal or posterior and injuring the nerve. In cases where the fibular head is not clearly palpable, the use of intraoperative radiographic imaging should be considered to correctly position the wire in both the sagittal and coronal planes.
